# Taking the rights of children with complex conditions seriously: New ethics challenges arisen during the COVID-19 pandemic

**DOI:** 10.3389/fped.2022.1001706

**Published:** 2022-09-26

**Authors:** Anna Zanin, Anna Santini, Enrico Furlan, Franca Benini

**Affiliations:** ^1^Palliative Care and Pain Service, Department of Women's and Children's Health, University of Padua, Padua, Italy; ^2^Research Group “Moral Philosophy and Bioethics”, Department of Molecular Medicine, University of Padua, Padua, Italy

**Keywords:** children's rights, COVID-19, complex care conditions, ethics, Paediatric Palliative Care Service

## Introduction

Children with complex care conditions (CCCs) and their families have always been a fragile population, at high risk of marginalisation and social exclusion, even prior to the outbreak of the COVID-19 pandemic. Few studies have explored in detail the impact of the pandemic on CCCs, and there are no shared guidelines on how to tackle the specific ethical dilemmas posed by the COVID-19 predicament. Both healthcare professionals and families improvised novel strategies to overcome the current crisis, but these tentative answers cannot be the solution in the long run.

In this article, we set out to highlight some new ethics challenges regarding CCCs arisen during the COVID-19 pandemic, referring on the one hand to the United Nations Convention on the Rights of the Child (UNCRC)^1^ and on the other hand to the experience of a Paediatric Palliative Care Service of the Veneto Region (Italy), presenting and discussing three real-life cases.

Clinical ethics considerations such as those inspired by Beauchamp and Childress' principlist approach (1) must be coupled with other relevant evaluations regarding the access to education (UNCRC, art. 28) or the protection of family unity (UNCRC, art. 9), without any discrimination (UNCRC, art. 23), and with a particular attention to children with special needs (UNCRC, art. 23). Furthermore, this lines up with Amartya Sen's approach (2), who identified the social opportunities of decent healthcare and education as prerequisites for developing one's capabilities and consequently for exercising one's freedom. The growing recognition of the importance of adopting a rights-centred approach in healthcare led the World Health Organisation (WHO) Europe to develop a set of tools to assess and improve the respect of children's rights also in primary healthcare^2^ (3).

Existing medical literature has shown that the COVID-19 pandemic has had a considerable impact on the paediatric healthcare settings (4–11). Social distancing and school closure, coupled with the reduction of other activities, have caused harmful psychological consequences on adolescents and the paediatric population in general (5, 6). Such negative consequences were especially heavy on CCCs, on minors with a migrant background, and with low socioeconomic status (7–10). In addition, visiting limitations during hospitalisation and reduction of elective healthcare activities have had long-term detrimental consequences (11, 12). In some specific situations, a delay in the interventions or very strict visiting policies have adversely influenced the care trajectory of these patients.

While it is important that paediatric teams take up their responsibilities to defend and promote these rights (4), it is also important to systematically collect the opinions and experiences of all parties involved (especially those of the minors and their parents) to accurately map their problems and to critically evaluate the balance that we have so far achieved between competing ethics concerns or competing rights. This seems a necessary preliminary step towards revising our priorities and better organising the services we deem essential for granting the basic rights of CCCs.

The three cases we have selected are meant to shed light on the kinds of ethics challenges we need to tackle through empirical research and philosophical reflection.

## Case 1: The story of Mary—The challenge of providing humane end-of-life care to CCCs even under strict public health measures to counter the pandemic

Mary was a 3-year-old girl, affected by gangliosidosis type 1. She was hospitalised in the COVID-19 Paediatric Emergency Department (ER) 1 week before her death, due to an acute respiratory distress syndrome caused by the COVID-19 infection. Her parents were not vaccinated. Her clinical neurologic condition had been evolving over time, and at the moment of hospitalisation, she presented with a progressive neurological impairment with increasing seizures, dystonic movements, and major difficulties in oral feeding. Pain and dyspnea management was challenging at the beginning of hospitalisation, with an improvement after the optimization of the analgesic therapy. Within 48 h, Mary presented a clinical evolution towards multiorgan failure. Since death was imminent, both parents were admitted to the unit, but regulations forbid other family members to visit her. This was a source of distress for Mary's mum and dad.

The family is of Macedonian origin. Mary's parents have been living in Italy for a long time. They had always wanted to bring the little girl to Macedonia, to introduce her to the rest of their large family. Unfortunately, their daughter's health problems, combined with the fear of contagion and closed borders, made the desired family reunion impossible.

The story of Mary and her family is emblematic of the difficulties to grant the respect of the child's right to spend time with all family members, also during hospitalisation in emergency times (UNCRC, art. 9), and to receive appropriate palliative care, including timely psychological support.

The ethics challenge we have to face in this case could be summarised as follows: how to balance the need to implement public health safety measures with the moral duty to provide humane end-of-life care to CCCs and to support their families?

## Case 2: The story of Federico—The challenge of granting effective educational opportunities to chronically ill patients

Federico is an 11-year-old boy, suffering from Duchenne's muscular dystrophy.

In spring 2020, Federico was in fifth grade, and due to the pandemic, the school attendance was disrupted and replaced by distance learning, which was somewhat disorganised at the beginning. After a few weeks, Federico's family was able to obtain that one educator would go to their house, but it was only for 1 h a week. All his pneumological, neurological, and cardiac follow-ups, as well as dental surgery, were postponed to a later date due to public health restrictions.

In September 2020, schools reopened for everyone but Federico. His parents were very anxious about the possibility of contagion. Furthermore, they were unconvinced to vaccinate their son and they decided not to send the boy to school. Federico always felt he had no say in the decision. Federico's school was very open to dialogue both with the boy's parents and with healthcare professionals, but at the same time it was not prepared to deal with Federico's fragile health situation during a pandemic crisis. Several meetings were held with the teaching staff to identify the best way to guarantee the boy's right to education and his desire to have social contacts while preserving his physical wellbeing.

For the first 2 months of the new school year, no educator was allowed to go to Federico's home, and only in November 2020, a home support teacher was finally assigned. He established a positive relationship with him, with good school performance. Contacts with classmates remained sporadic.

The story of Federico is emblematic of the difficulties to defend CCCs' right to give their opinion on issues affecting them (UNCRC, art. 12) and to grant CCC's right to education and social interaction even if seriously ill (UNCRC, art. 28).

The ethics challenges we have to face in this case could be summarised as follows: how to balance the understandable desire to reduce the probability of contagion in fragile patients with the crucial need to grant access to effective education and social interaction, which are instrumental in the flourishing of CCCs? How to include CCCs who are cognizant of the situation in decision-making regarding their education?

## Case 3: The story of Alex—The challenge of granting access to adequate elective care for cancer patients

Alex was a 10-year-old patient with anaplastic ependymoma. He was hospitalised to receive radiotherapy (RT) in order to contain the symptoms. After 10 days, he contracted the SARS-COV2 infection in the hospital and had to be isolated (together with his mother) in the paediatric COVID-19 ward. Alex's family originates from East Africa, and his mother did not speak Italian, making communication *via* standard online devices very difficult. In the new ward, Alex did not feel comfortable: all the doctors and nurses were dressed in white, their faces masked, and their bodies completely covered by protective equipment. It was not easy for him to understand who they were and what they wanted to do on him. His mother found it even harder to understand what was happening and fell into a depression.

Since he was infected with SARS-CoV-2, Alex's RT was scheduled in the late evening, without the possibility for him to be accompanied by anyone. Consequently, he was terribly anxious and experienced a crisis of psychomotor agitation during one of the RT sessions. For this reason, the clinical staff decided to suspend the RT till the resolution of the SARS-CoV-2 infection, which lasted 20 days. Once retransferred to the standard hospitalisation unit, due to the positivity of his caregiver, he needed to be isolated again in the COVID unit for 5 more days, with a new suspension of the RT. Eventually, since he had become depressed as a result of the prolonged isolation, it was decided to send him back home. The subsequent RT sessions were organised with daily transport from his home to the hospital and back. Unfortunately, shortly afterward Alex passed away.

The story of Alex is emblematic of the difficulties to grant the right of CCCs to access treatments in a timely manner (UNCRC, art. 6), the right not to be separated from their family (UNCRC, art. 9), and the right to rest, relax, play, and take part in creative activities (UNCRC, art. 31).

The ethics challenge we have to face in this case could be summarised as follows: how to balance the necessity to reorganise and prioritise the healthcare resources during a pandemic with the importance to ensure vital access to elective care (such as cancer treatments)?

## Discussion and conclusion

The ethics challenges we have presented are just a sample of the new ethics issues arisen in the care of CCCs and their families during the current COVID-19 pandemic.

While the literature has focused so far on the ethics dilemmas connected with the drastic restriction of visitation policy in acute settings (11, 12), especially during the first waves, less attention has been dedicated to the impact of the reorganisation of the healthcare services and to the repercussions of public health policies on some fundamental rights of children with complex conditions. Indeed, many CCCs have experienced a remarkable reduction in the enjoyment of basic rights, such as access to timely therapies, access to education, and the possibility to socialise, play, and take part in cultural and creative activities.

At the beginning of the pandemic, some families of CCCs reported that social isolation was perceived as a form of protection (13, 14). Later on, however, both parents of CCCs and healthcare providers were confronted with a new kind of ethics dilemmas: what is the right balance between the duty to protect and preserve health and the duty to grant children the social and cultural experiences that are essential to their flourishing and to their mental wellbeing? How can we put in place the necessary public health policies without compromising the access to therapies to particularly fragile children and families? The solutions devised by some healthcare institutions, that were able to reorganise their services in order to offer home care, are commendable and a sign of moral creativity, i.e., of the tension to constantly find new ways to interpret and implement moral values, especially in response to unprecedented situations. For instance, in the case of Alex, the healthcare team was able—alas only in his last days—to organise a daily transport from home to the hospital, in order to grant both access to needed therapy and the crucially important closeness to the family. However, more structured prevention measures and the definition of new policies require a preliminary effort to carry out research involving all stakeholders and more nuanced moral reflection. Indeed, only after such a process we could tackle the issue, for example, of how to balance the need for healthcare professionals to wear personal protective equipment and the importance not to scare already fragile and confused minors.

This is why we end this article by calling for widespread mix-methods studies on the experiences and opinions of CCCs, families, educators, and healthcare providers. Patient-reported outcomes (PROs) (15, 16) should be more integrated in clinical research as they provide information on how healthcare affects patient health and wellbeing in a patient- and family-centred approach.

Coupling ample stakeholders' involvement with frank moral discussion seems to us the best way to tackle the thorny ethics issues we have highlighted, such as the balance between the rights of the single and the community or the balance in resource allocation of our already stressed healthcare systems.

## Author contributions

AZ conceived the article. AZ and AS collected and described the clinical cases and drafted the first version of the manuscript. EF thoroughly revised the article with specific attention to the ethics considerations. FB supervised this work and discussed with all the authors the clinical cases and the overview of the topic. All authors discussed, contributed, and approved the final manuscript.

## Conflict of interest

The authors declare that the research was conducted in the absence of any commercial or financial relationships that could be construed as a potential conflict of interest.

## Publisher's note

All claims expressed in this article are solely those of the authors and do not necessarily represent those of their affiliated organizations, or those of the publisher, the editors and the reviewers. Any product that may be evaluated in this article, or claim that may be made by its manufacturer, is not guaranteed or endorsed by the publisher.

## Footnotes

^1^ https://www.unicef.org/child-rights-convention/convention-text-childrens-version

^2^ https://ec.europa.eu/info/aid-development-cooperation-fundamental-rights/your-rights-eu/eu-charter-fundamental-rights_en
